# Onset of frequent dust storms in northern China at ~AD 1100

**DOI:** 10.1038/srep17111

**Published:** 2015-11-26

**Authors:** Yuxin He, Cheng Zhao, Mu Song, Weiguo Liu, Fahu Chen, Dian Zhang, Zhonghui Liu

**Affiliations:** 1 School of Earth Sciences, Zhejiang University, Hangzhou, 310027, People’s Republic of China; 2 Department of Earth Sciences, The University of Hong Kong, Hong Kong, People’s Republic of China; 3 State Key Laboratory of Lake Science and Environment, Nanjing Institute of Geography and Limnology, CAS, Nanjing, 210008, People’s Republic of China; 4 State Key Laboratory of Loess and Quaternary Geology, IEE, CAS, Xi’an, 710075, People’s Republic of China; 5 Key Laboratory of Western China’s Environmental Systems, School of Earth and Environment Sciences, Lanzhou University, Lanzhou, 73000, People’s Republic of China; 6 Department of Geography, The University of Hong Kong, Hong Kong, People’s Republic of China

## Abstract

Dust storms in northern China strongly affect the living and health of people there and the dusts could travel a full circle of the globe in a short time. Historically, more frequent dust storms occurred during cool periods, particularly the Little Ice Age (LIA), generally attributed to the strengthened Siberian High. However, limited by chronological uncertainties in proxy records, this mechanism may not fully reveal the causes of dust storm frequency changes. Here we present a late Holocene dust record from the Qaidam Basin, where hydrological changes were previously reconstructed, and examine dust records from northern China, including the ones from historical documents. The records, being broadly consistent, indicate the onset of frequent dust storms at ~AD 1100. Further, peaked dust storm events occurred at episodes of high total solar irradiance or warm-dry conditions in source regions, superimposed on the high background of frequent dust storms within the cool LIA period. We thus suggest that besides strong wind activities, the centennial-scale dust storm events over the last 1000 years appear to be linked to the increased availability of dust source. With the anticipated global warming and deteriorating vegetation coverage, frequent occurrence of dust storms in northern China would be expected to persist.

Dust storms are one of the severe natural disasters that frequently occur in northern China^1^,^2^. Dust aerosols have critical influences on climatology, biogeochemistry and human health^1^,^2^,^3^,^4^. For instance, airborne dust particles could cause serious damage to vegetation and crops by abrasion^3^. Inhalation of dust could cause respiratory illnesses such as silicosis, asthma, bronchitis and chronic obstructive pulmonary disease^4^. The Qaidam Basin is responsible for up to 50% of dust to the Chinese Loess Plateau^5^,^6^. Hence studies of dust activities in the Qaidam Basin are important to understanding the atmospheric dust flux across mid-latitude China.

The magnitude of dust storm intensity is influenced by the combination of wind strength and hydrological conditions in source regions, since the formation of dust storm requires dust source, strong wind and low surface vegetation coverage^7^. Firstly, intensified cold air cyclonic activities across large areas, i.e. Siberian High, during cool periods might increase dust emission^8^,^9^. Stronger dust storms in northern China are suggested to have occurred in the cool Little Ice Age^7^,^10^ (LIA, AD 1400-1850). Meanwhile, dust storms in the arid region tend to be easily activated due to low regional effective moisture and lack of vegetation cover^7^,^9^. Thus both global and regional climates could strongly influence dust storm frequency and intensity.

Many archives have been applied for dust storm investigations. For instance, meteorological records could precisely reveal dust storm information at high resolution. However, meteorological data in northwestern China are only available since ~AD 1950 (ref. 11). Chinese historical documents could reveal the dust storm frequency in northern China over the past 1700 years with accurate chronology^12^,^13^, but these documents are mainly from eastern China instead of the western part, the dust source regions. Further, dust events might be underestimated toward earlier period due to fewer documents available. Grain-size distribution in loess deposits is useful for reconstructing eolian circulation pattern^14^. Sediments from hydrologically-closed lakes in arid regions have also been proposed as ideal archives for dust storm studies^10^,^15^,^16^. Lakes in the Qaidam Basin are mostly small and hydrologically-closed, since they are mostly fed by groundwater due to low precipitation and surface runoff. Coarse particles are carried to lake center by strong winds in winter/spring time. Since their thermal capacity is less than that of ice, the coarse particles could be preserved on the ice surface as the ice beneath them would melt first, and deposit in the lake bottom after ice melts^10^,^15^. Thus the coarse fraction can be used as an excellent proxy for past eolian dust variability in the region.

Here we investigate the dust storm history in northern China at decadal resolution over the late Holocene by analyzing grain size distributions from a hydrologically closed lake, Lake Gahai (Fig. 1, Supplementary Fig. S1), where hydrological changes were previously reconstructed from the same core^17^. We first establish that the dust storm record from Lake Gahai, within chronological uncertainty, is broadly consistent with other records in mid-latitude China^10^,^16^,^18^,^19^,^20^,^21^,^22^,^23^,^24^, indicating the onset of frequent dust stroms at ~AD 1100. With multiple proxy records generated in the same core from Lake Gahai, we then associate detailed dust frequency changes with regional climatic and hydrological conditions, unaffected by chronological uncertainty. This association is further substantiated with comparison between the historical dust record and total solar irradiance changes^25^,^26^ (TSI), for which high TSI corresponding to warm/dry conditions in arid northwestern China was identified previously^17^,^27^. Such close inspections allow us to attribute the dust storm variability to both the strength of Siberian High and regional hydrological changes, with the later linked to vegetation coverage and dust source availability.

## Results

### Grain-size based dust storm record from Lake Gahai

Grain particles through the Lake Gahai core can be divided into three major assemblages: finer (0–10 μm), median (10–63 μm) and coarser (>63 μm) sub-populations (Fig. 2a,c). The mean grain size values range from 8 to 125 μm. In samples with larger grain size values, the main particle fraction peaks at coarser sub-population, with a secondary peak centered at finer sub-population (e.g. sample at AD 1631, coarser peak at 177 μm, while finer peak at 6 μm, Fig. 2c). Meanwhile, samples with smaller mean grain size mainly contain particles smaller than 10 μm (e.g. AD 998, Fig. 2c). Prior to ~AD 1100, the >63 μm fraction was close to 0% at most of time, occasionally reaching to ~10% at ~250 BC and 50 BC–AD 250 (Figs 2b and 3g). Since ~AD 1100, the >63 μm fraction increased dramatically, up to 62%, and substantial fluctuations persisted, indicating a regime shift in dust storm frequencies.

### Synthesized dust storm record across the mid-latitude Asia

Considering the chronological uncertainties, the onset of intensive dust storms at ~AD 1100 inferred from Lake Gahai can be corraborated by various proxy records^10^,^16^,^18^,^19^,^20^,^21^,^22^,^23^,^24^ (Supplementary Fig. S2, Supplementary Table S1). Fristly, our record from Lake Gahai shows a similar pattern with the records from lakes in and near the Qaidam Basin^10^,^18^,^19^, the Xinjiang region^20^, and to the further west, the Aral Sea in the central Asia^21^. Besides lake sediments, dust contents in Guliya^22^ and Dunde^23^ ice cores also roughly support the dust storm pattern from Lake Gahai. Dust records of the varved lacustrine sediments from Lake Xiaolongwan and Lake Sihailongwan in northeastern China^16^ agree well with ours too. This pattern might be further extended to off-shore area, such as Cheju Island^24^. This poses a connection between the dust source areas (the central Asia) and the downwind dust deposition sites (eastern China) via long distance transportation through the atmosphere.

To minimize the inevitable chronological errors among the records discussed above, all proxy records were synthesized to represent large-scale dust storms in the mid-latitude Asia over the past 2000 years (Methods, Supplementary Fig. S2). The 50-year averaged synthesized curve clearly indicates onset of frequent dust storms at ~AD 1100 (Fig. 3f). The proxy-based curve is also broadly consistent with the records of dust storms compiled from Chinese historical literatures (Fig. 3e), with a better constrained chronology. Therefore, the onset of frequent dust storms at ~AD 1100, within the warm Medieval Warm Period^28^ (MWP, Fig. 3b), appears to be a robust feature.

## Discussion

Stronger dust storms prevailing in the LIA with a peak at ~AD 1500 have been suggested in many studies^7^,^10^. The overall frequent dust storms within the relatively cool LIA period could be linked to intensified Siberian High as inferred by the non-sea salt potassium (nssK^+^) content from Greenland ice core^29^ (Fig. 3c), suggesting an important role of the Siberian High strength in dust storm variations. The Siberian High anticyclone over Eurasia is maximized in April, synchronous with dust storms^30^. Intensified cyclonic activities in cooler periods might strengthen the invasion of cold air from Siberia, and increase the dust emission in this arid region^8^,^9^.

However, the onset occurring within the MWP and the peaked dust events during relatively warm episodes within the LIA, are difficult to be explained by the intensified Siberian High alone (Fig. 4). Confidently identified by associations with temperature and salinity records from the same core^17^, high dust input in Lake Gahai occurred at centennial warm and dry episodes (Fig. 4). One might argue that increased coarse fraction in Lake Gahai could also be caused by relatively low lake level during dry episodes, not necessarily intensified dust storms. However, overall increased coarse fraction occurred during the wet LIA with high lake level. Even at those warm episodes within the LIA, when much higher coarse fraction occurred, lake level was probably not as low as before AD 1100. Indeed, before AD 1100, occasionally increased coarse fraction mostly occurred at relatively cool/wet periods (Supplementary Fig. S3). These all suggest the >63 μm fraction is not strongly affected by lake level variations.

To further substantiate this association, we also compared the dust records derived from historical documents^12^,^13^ with TSI changes^25^,^26^ (Fig. 4b). In the historical records, major peaked dust events occurred at episodes centered at ~AD 1200, 1600 and 1800, all corresponding to high TSI episodes (Fig. 4b), when relatively warm-dry conditions occurred in northwestern China^17^,^27^. The two independent approaches thus confirm that at centennial scales, the peaked dust events, including the onset at ~AD 1100 (Fig. 3), occurred at warm episodes, which we attributed to increased availability of dust source, together with the overall intensified wind field for dust transportation. During warm episodes associated with high TSI, increased regional evaporation and reduced rainfall^17^ would lower effective moisture thus deteriorate vegetation cover in the arid central Asia. Less vegetation cover would lead to higher soil erosion, less wind reduction and less dust particles trapped^7^,^9^, and increase the availability of dust sources.

Our inference could further be corroborated with the comparison of dust records with the Siberian High reconstructed from Greenland ice core^29^. The timing of peaked dust storms reconstructed from Chinese historical documents^12^,^13^, and by inference from Lake Gahai, seems to be non-synchronous to the Siberian High at centennial scales (Fig. 4). Peaked dust events in China occurred at high TSI episodes (Fig. 4b), while the Greenland terrestrial dust peaked at low TSI episodes (Fig. 4a). This suggests that the Greenland record reflects more the capacity of long-distance, planetary transportation, thus more related to the Siberian High, while dust storms in central Asia, including the downwind East Asia, were additionally affected by the dust source availability. If correct, it also explains that the onset of frequent dust storms at ~AD 1100 within the MWP, when the Siberian High was not particularly intensified, was not recorded at Greenland (Fig. 4a).

However, why the onset started at this particular time remains unclear as warm-dry conditions, if not warmer or drier, frequently occurred earlier. There existed little agricultural activity in northwestern China due to harsh living environments. Despite slight increase during the MWP, the population there were generally <10 million before AD 1700 (Fig. 3d, ref. 31). Considering the population size and technologies used then, anthropogenic human impacts on the vegetation coverage would be secondary, as compared to natural climate variability. Plausibly, extended warm and dry conditions during the MWP in source regions would have severely deteriorated vegetation coverage and thus triggered the onset of frequent dust storms at ~AD 1100, while the gradual intensification of the Siberian High toward the LIA provided necessary dynamical conditions for strong/frequent dust activities.

In summary, our close inspection on dust records revealed the onset of frequent dust storms in northern China at ~AD 1100 during the warm/dry MWP and detailed dust variations within the cool/wet LIA, which are difficult to be explained by the intensified Siberian High alone. We deciphered two factors that could impact dust storm variations in northern China. During the cool/wet LIA, overall frequent dust storms were associated with the intensified Siberian High^7^,^10^. Superimposing peaked dust events at centennial warm/dry episodes could be linked to reduced effective moisture and deteriorated vegetation coverage in source regions. Our study indicates that even under natural conditions, dust storms in northern China could become more frequent due to the increased availability of dust sources in a warm climate. With the anticipated global warming and increasing human activities in the region, largely adverse to vegetation coverage, frequent occurrences of dust storms would thus be expected to persist in northern China.

## Methods

### Location

**Figure 1:**
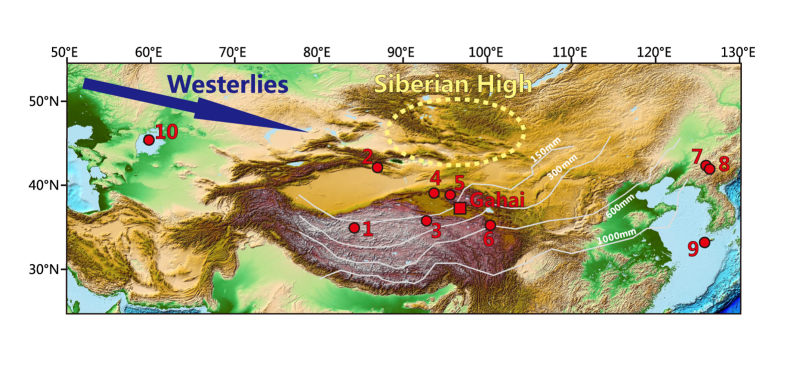
Overview map showing the study site, Lake Gahai (square) and sites of other dust records discussed here (circles). 1: Guliya ice core^22^; 2: Lake Bosten^20^; 3: Lake Kusai^18^; 4: Lake Sugan^10^; 5: Dunde ice core^23^; 6: Lake Gengga^19^; 7: Lake Sihailongwan^16^; 8: Lake Xiaolongwan^16^; 9: Cheju Island^24^; 10: Aral Sea^21^. The map was generated using ESRI ArcGIS v9.3 software with SRTM DEM database from Geospatial Data Cloud (http://www.gscloud.cn) shared by Computer Network Information Center, Chinese Academy of Sciences. Annual rainfall isohyets in China, Siberian High and westerlies are also indiacted in the map.

**Figure 2:**
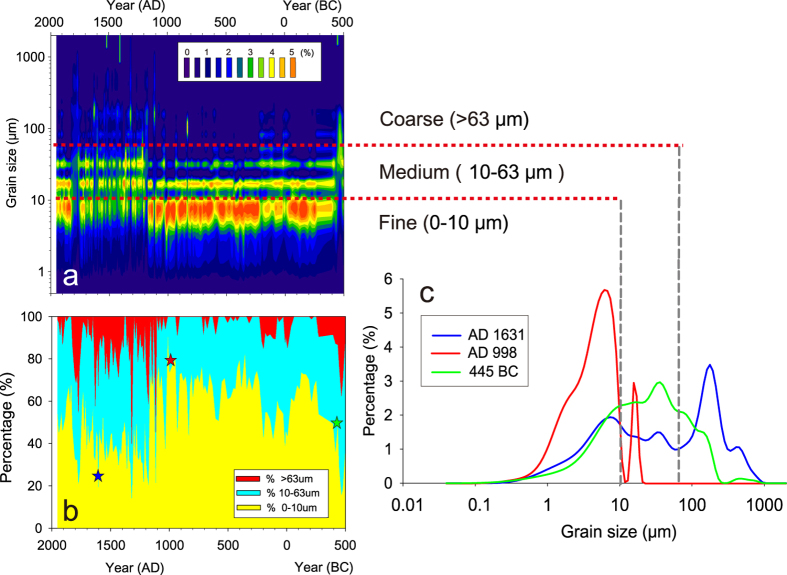
Grain size distribution in sediments from Lake Gahai over the last 2500 years. (**a**) Contour plot of grain size distribution. (**b**) Percentage of three major grain assemblages: fine (0–10 μm), medium (10–63 μm), and coarse (>63 μm) sub-populations. (**c**) Grain size distributions in three representative samples of Lake Gahai sediments, indicated by stars in (**b**).

Lake Gahai (37°8′ N, 97°31′ E, 2848 m a.s.l., Fig. 1, Supplementary Fig. S1) is located at the eastern edge of the Qaidam Basin on the northeastern Tibetan Plateau. Most of the basin area is covered by gobi, deserts, and playas. The average elevation of the basin is 2800 m a.s.l., while the surrounding mountains rise to elevations of ~5000 m a.s.l. To the west lies Lake Hurleg and Lake Toson, which connect Lake Gahai with alluvial fans. The current Lake Gahai area is 35 km^2^ with a maximum water depth of 15 m and mean depth of 8 m. Water in Lake Gahai is of Na-Mg-SO_4_ type, with a pH value of 8.3 (ref. 32) and a salinity of ~90.6 g/L (ref. 33). Mean annual temperature at the nearby Delingha meteorological station is 4 °C and mean annual precipitation is about 160 mm (falling mostly during the summer), while the potential evaporation is about 2000 mm.

### Chronological profile

A 2.5 m lake sediment core (QHC09-4) from Lake Gahai was retrieved in the summer of 2009. Based on the excess ^210^Pb results on the topmost sediments and the cross-comparison of two similar dry events in the upper part of the salinity records from Lake Gahai and Lake Sugan^17^, the top ~50-year sediments in the Gahai core were assumed to be missing during the coring process. Chronology of core QHC09–4 was then established by 4 AMS-^14^C dates on bulk organic matters through the core (Supplementary Fig. S1). AMS-^14^C dates were calibrated to calendar ages using the CALIB Rev 6.0.1 calibration program^34^, after correction of reservoir ages of 1855 ^14^C years based on regression method. Detailed information on the age profile can be found in ref. 17.

### Grain-size analysis

Core QHC09-4 was subsampled with every 0.5 cm slice, whereas samples of 1 cm interval for the top 90 cm and ~2 cm for the rest of core were taken for grain size analysis according to the methods described by Konert and Vandenberghe^35^. The freeze-dried samples (~1 g) were pretreated with hot hydrogen peroxide (10% H_2_O_2_, ~80 °C) and hydrochloric acid (1 mol/L HCl, ~80 °C) to remove organic matters and dissolvable salts, with the remains generally representing the size of terrestrial debris. The pH value of the solution of residual sample was then adjusted to 7 by repeatedly rinsing samples with distilled water. After ultrasonic pretreatment with the addition of sodium metaphosphotate [(NaPO_3_)_6_] solution in order to disperse the particles, the grain size of samples was measured by Malvern Mastersizer 2000 s laser diffraction particle analyzer, which has a measurement range of 0.04–2000 μm.

### Synthesized dust storm record across the mid-latitude Asia over the past 2000 years

**Figure 3:**
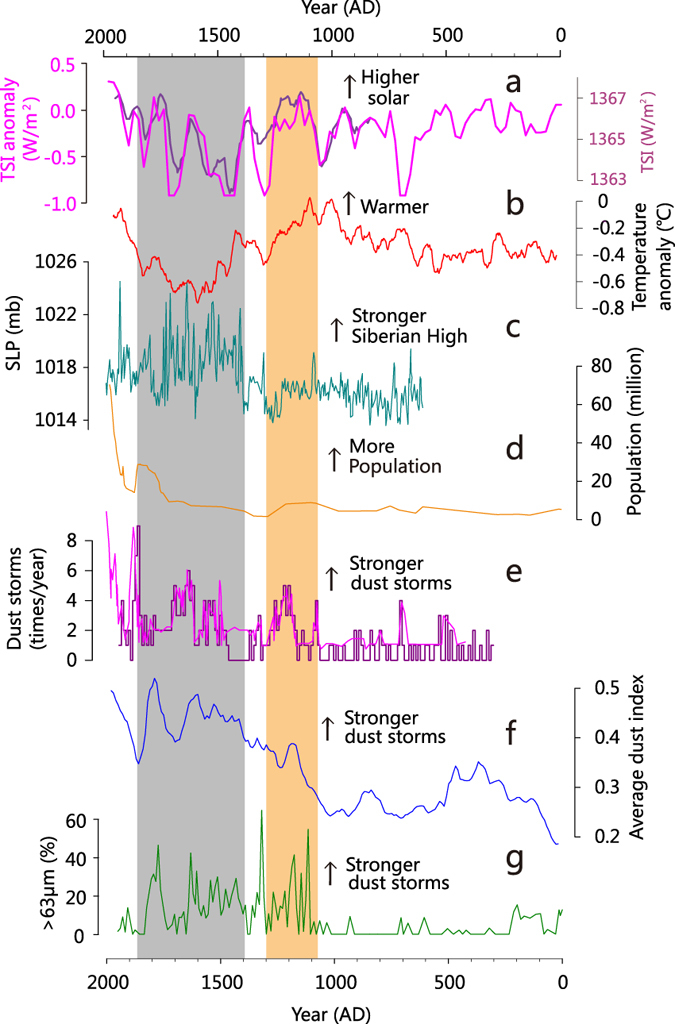
Variations in dust storm events in northern China compared with global and regional conditions. (**a**) Reconstructed TSI (purple line is from ref. 25 whereas pink line from ref. 26). (**b**) Northern Hemisphere mean temperature anomaly^28^. (**c**) Reconstructed Siberian High strength^29^. (**d**) Total population of five provinces in northwestern China (Shanxi, Gansu, Ningxia, Qinghai, Xinjiang)^31^. (**e**) Historical dust storm frequency records from northern^12^ and eastern^13^ China. (**f**) The 50-year averaged synthesis dust storm record across the mid-latitude Asia from Supplementary Fig. S2. (**g**) Percentage of >63 μm particles from Lake Gahai. Grey shading indicates the cool LIA period while orange indicates the early onset of frequent dust storms at ~AD 1100 within the warm MWP.

**Figure 4:**
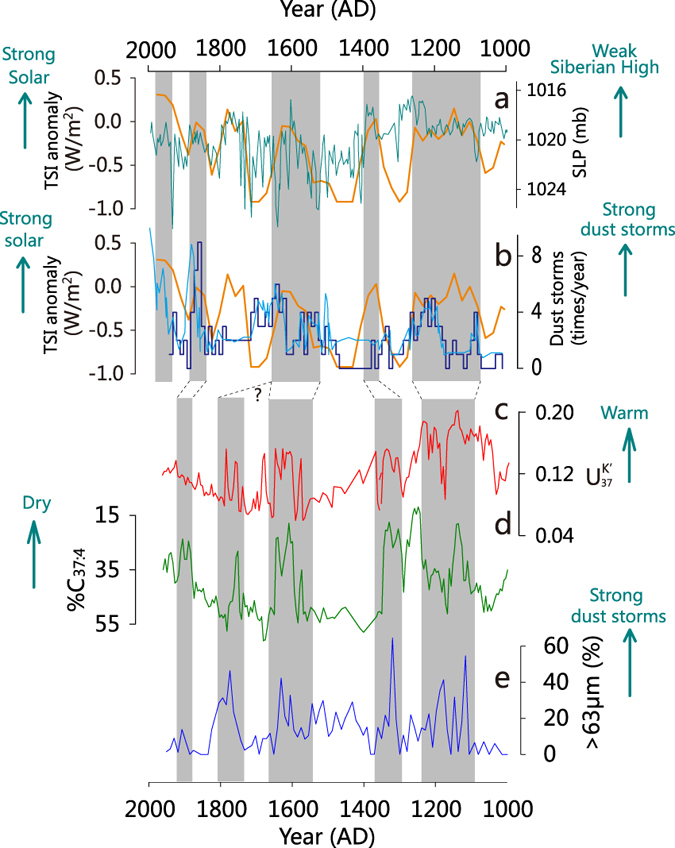
Association of dust storm events with solar irradiance and regional climatic records over the past 1,000 years. (**a**) Reconstructed Siberian High strength^29^ and (**b**) Historical dust storm records from China^12^,^13^, both superimposed with the reconstructed TSI anomaly^26^. Note the sea level pressure plotted inversely in (**a)**. (**c**) Alkenone-based 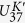-temperature record from Lake Gahai^17^. (**d**) Alkenone-based %C_37:4_-salinity record from Lake Gahai^17^. (**e**) Percentage of >63 μm particles from Lake Gahai. Periods of peaked dust storm events in northern China were highlighted with grey shadings. The overall increased dust activities within the cool/wet LIA were indicated with a dashed line.

Grain size records generated from sediment cores retrieved from the center of lakes were selected for this study. The chronologies and the fractions to represent dust variability followed the original publications. To minimize the bias deriving from the uncertainty of the dust proxy, the influence of local hydrology on the large-scale dust storm and the inevitable chronological errors among the records, all the selected dust storm proxy records (Supplementary Fig. S2, Supplementary Table S1) were linearly interpolated to a uniform 10-year interval, standardized to have a mean value of 0.5 and variation of 1. The synthesized dust storm sequence was produced by arithmetically averaging all of the standardized high-resolution dust storm time series (Supplementary Fig. S2). Finally, a 50-year (5-point) average sequence of dust storm strength across the mid-latitude Asia was presented in Fig. 3f.

## Additional Information

**How to cite this article**: He, Y. *et al.* Onset of frequent dust storms in northern China at ~AD 1100. *Sci. Rep.* **5**, 17111; doi: 10.1038/srep17111 (2015).

## Supplementary Material

Supplementary Information
