# Supplementary material for: Onset of frequent dust storms in northern China at ~AD 1100
Source: Sci Rep. 2015 Nov 26;5:17111. doi: 10.1038/srep17111 (PMC4660819; doi:10.1038/srep17111)
Supplement: Supplementary Information [file srep17111-s1.pdf]

Supplemental Information

Onset of frequent dust storms in northern China at ~AD 1100

Yuxin He, Cheng Zhao, Mu Song, Weiguo Liu, Fahu Chen, Dian Zhang & Zhonghui Liu

A. Supplemental Table

Table S1| Information of dust storm proxy records described in the main text.

B. Supplemental Figures

Figure S1| Coring site and chronological profile of the Lake Gahai core.

Figure S2| Comparative diagrams of proxy-based dust storm variations across the mid-latitude Asia. a, Grain-size based dust record from the Aral Sea. **b,** Grain-size based dust record from Lake Bosten. **c,** Dust content from Guliya ice core. **d,** Percentage of particles with diameter >63 μm from Lake Sugan. **e**, Percentage of particles with diameter >63 μm in sediments from Lake Gahai (this study). **f,** Grain size HI index from Lake Kusai. **g,** Percentage of particles with diameter >63 μm in sediments from Lake Gengga. **h,** Dust content from Dunde ice core in the Tibetan Plateau. **i,** Variation of minerogenic clastic content of Lake Sihailongwan sediments. **j,** Variation of minerogenic clastic content of Lake Xiaolongwan sediments. **k,** Coarse grain record from Cheju Island. **m,** The synthesis dust storm records based on the average of standardized records from **a** to **k**. The number of proxy records used to generate the curve in each period. Strong dust events are highlighted with grey shadings.

Figure S3| Comparison of dust input variations with climatic records generated from the same core in Lake Gahai, and solar irradiance changes, over the past 2500 years. a, The reconstructed total solar irradiance (TSI) records. **b,** Alkenone-based $U_{37}^{K'}$- temperature record from Lake Gahai. **c,** Alkenone-based $\%C_{37:4}$-salinity record from Lake Gahai. **d,** Percentage of particles with diameter >63 μm in sediments from Lake Gahai. Strong dust events, corresponding to cool/wet conditions, are highlighted with grey shadings, while peaked dust events associated with warm/dry periods, since ~AD 1100, are highlighted with brown shadings. The TSI curve is plotted for reference as direct comparison between TSI and proxy records is difficult due to chronological uncertainties.

Table S1| Information of dust storm proxy records described in the main text.

No.	Site	Location	Archive	Proxy used	References
	Lake Gahai	37°8’N, 97°31’E	Sediment	Grain size (>63μm)	This study
1	Guliya	35°17'N, 81°29'E	Ice core	Dust content	22
2	Lake Bosten	42°5'N, 87°3'E	Sediment	Grain size (>63μm)	20
3	Lake Kusai	35°44’N, 93°27’E	Sediment	Grain size (HI index)	18
4	Lake Sugan	38°52'N, 93°54'E	Sediment	Grain size (>63μm)	10
5	Dunde	38°6'N, 96°24'E	Ice core	Dust contents	23
6	Lake Gengga	36°11’N, 100°6’E	Sediment	Grain size (>63μm)	19
7	Lake Sihailongwan	42°18'N, 126°21'E	Sediment	Clastic content	16
8	Lake Xiaolongwan	42°17'N, 126°36'E	Sediment	Clastic content	16
9	Cheju Island	31°45'N, 125°45'E	Sediment	Grain size (PCA component)	24
10	Aral Sea	40°0'N, 62°30'E	Sediment	Grain size (fraction ratio)	21
11	Northern China		Historical documents	Dust storm events	12, 13

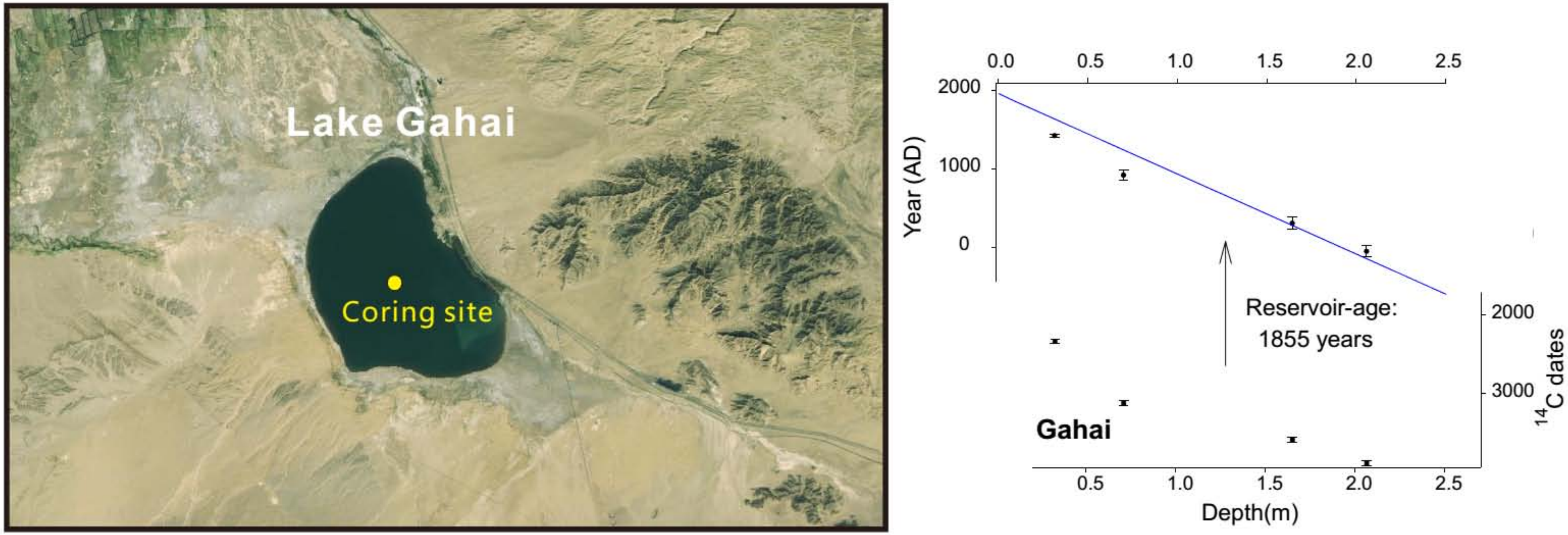

Figure S1| Coring site and chronological profile of the Lake Gahai core[17].

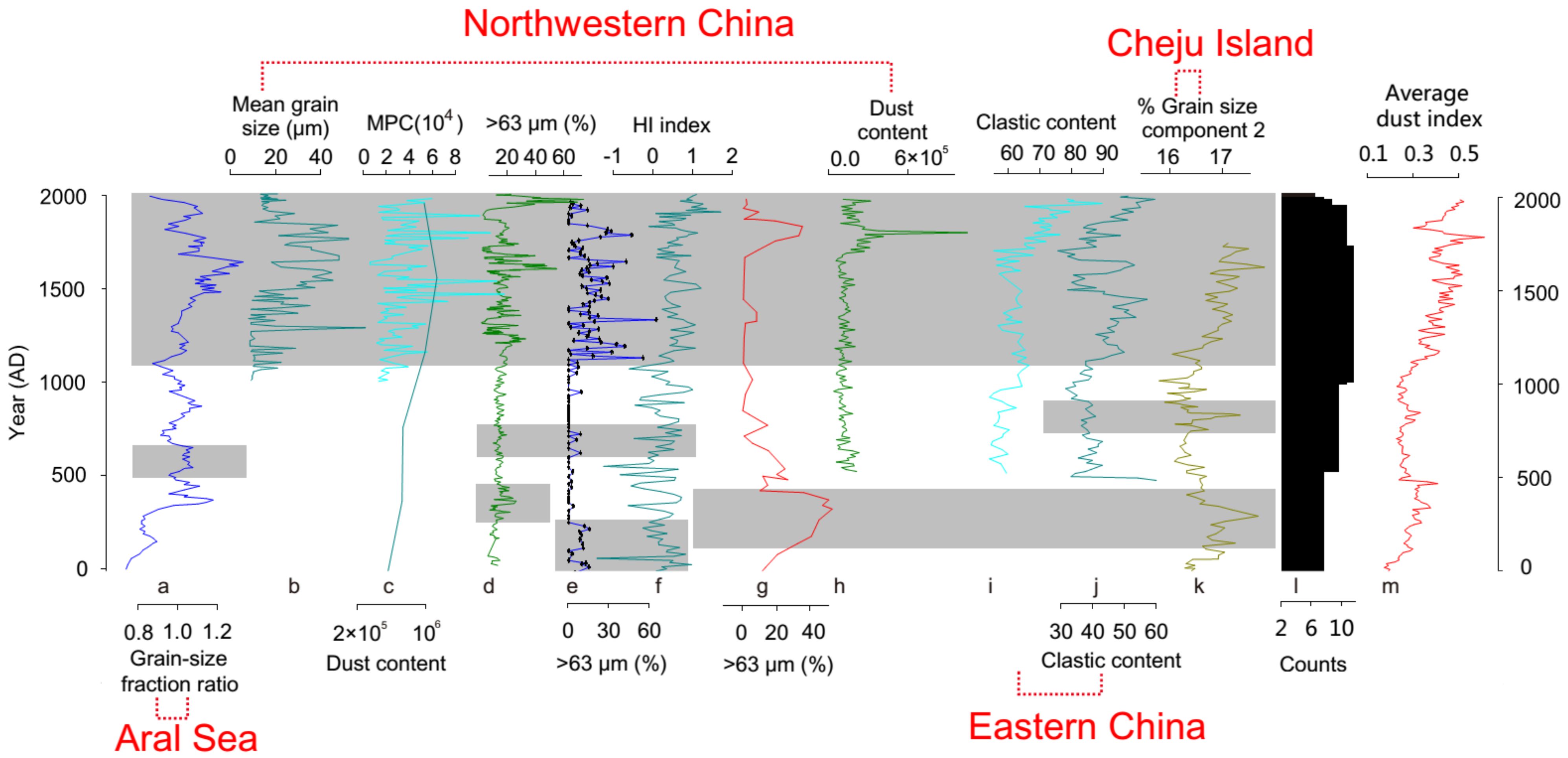

Figure S2| Comparative diagrams of proxy-based dust storm variations across the mid-latitude Asia. a, Grain-size based dust record from the Aral Sea[21]. **b,** Grain-size based dust record from Lake Bosten[20]. **c,** Dust content from Guliya ice core[22]. **d,** Percentage of particles with diameter >63 μm from Lake Sugan[10]. **e**, Percentage of particles with diameter >63 μm in sediments from Lake Gahai (this study). **f,** Grain size HI index from Lake Kusai[18]. **g,** Percentage of particles with diameter >63 μm in sediments from Lake Gengga[19]. **h,** Dust content from Dunde ice core in the Tibetan Plateau[23]. **i,** Variation of minerogenic clastic content of Lake Sihailongwan sediments[16]. **j,** Variation of minerogenic clastic content of Lake Xiaolongwan sediments[16]. **k,** Coarse grain record from Cheju Island[24]. **m,** The synthesis dust storm records based on the average of standardized records from **a** to **k**. The number of proxy records used to generate the curve in each period. Strong dust events are highlighted with grey shadings.

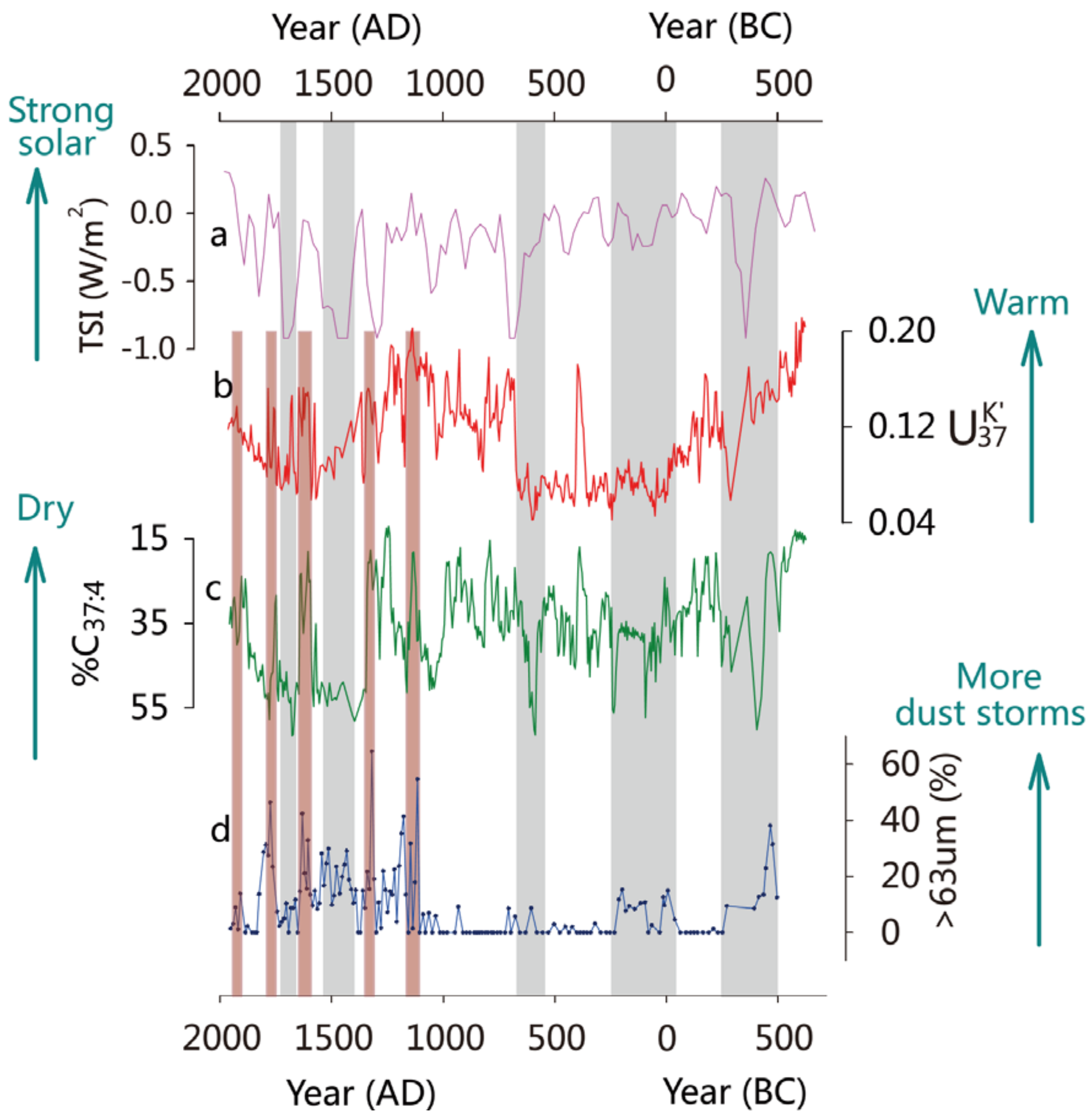

Figure S3| Comparison of dust input variations with climatic records generated from the same core in Lake Gahai, and solar irradiance changes, over the past 2500 years. **a,** The reconstructed total solar irradiance (TSI) records[25, 26]. **b,** Alkenone-based $U_{37}^{K'}$- temperature record from Lake Gahai[17]. **c,** Alkenone-based %$C_{37:4}$-salinity record from Lake Gahai[17]. **d,** Percentage of particles with diameter >63 μm in sediments from Lake Gahai. Strong dust events, corresponding to cool/wet conditions, are highlighted with grey shadings, while peaked dust events associated with warm/dry periods, since ~AD 1100, are highlighted with brown shadings. The TSI curve is plotted for reference as direct comparison between TSI and proxy records is difficult due to chronological uncertainties.
